# A case report of brain abscess caused by *Nocardia cyriacigeorgica* in a diabetic patient

**DOI:** 10.1099/jmmcr.0.005133

**Published:** 2018-01-10

**Authors:** Mehdi Khorshidi, Sepehr Navid, Davood Azadi, Darioush Shokri, Hasan Shojaei

**Affiliations:** ^1^​ Department of Microbiology, School of Medicine, Isfahan University of Medical Sciences, Isfahan, Iran; ^2^​ Infectious Diseases and Tropical Medicine Research Center, Isfahan University of Medical Sciences, Isfahan, Iran

**Keywords:** brain abscess, diabetic foot ulcer, *Nocardia cyriacigeorgica*, 16S rRNA

## Abstract

**Introduction:**

*Nocardia* are Gram-positive partially acid-fast bacilli capable of inducing a wide range of infections in patients with immunodeficiency, AIDS, cancer, lupus erythematous and diabetes. *Nocardia cyriacigeorgica* was first isolated in 2001 from a patient with chronic bronchitis. Since then, there have been reports on the clinical significance of this organism in patients with bronchitis, brain abscess and lung diseases. We, herein, report a case of brain abscess in an elderly diabetic patient from Iran.

**Case presentation:**

The patient was a 73 year-old woman admitted to hospital due to severe headache and shortness of breath. The patient had lived with diabetes for 20 years and suffered from chronic foot ulcer. She was admitted to hospital with fever, weakness, drowsiness and vomiting. Clinical examination and the head CT scan of the left frontal lobe of the brain revealed a metastatic carcinoma involving skull bone in the tumor that resulted in two surgical operations in the following two years. The brain abscess biopsy revealed an infection with *Nocardia cyriacigeorgica* confirmed by phenotypic and molecular tests including a PCR-based amplification of a target genetic marker, a 596 bp fragment of 16S rRNA gene, followed by almost full 16S rRNA sequencing.

**Conclusion:**

The rare infections, such as brain abscess with *Nocardia,* are easily neglected or misdiagnosed due to the fastidious nature of the organism and inadequate microbiological experience of laboratories in the hospitals of developing countries. This case shows that hospitals should consider a better laboratory protocol to deal with the clinical cases in which fastidious organisms, and in particular *Nocardia*, are involved.

## Introduction

Nocardia was first described by Nocard in 1888 as a fungus but was later classified as an aerobic bacterium and member of the genus *Nocardia*, and order *Actinomycetales* [1]. *Nocardia cyriacigeorgica* is a Gram-positive, non-motile, spore-less, aerobic, partially acid-fast bacillus that is a member of the actinomycetes It was first isolated by Yasin *et al.* in 2001 from a chronic bronchitis patient [2]. This bacterium could be mainly found in soil but it can also live in still water and enter the human body through dust respiration and traumatic inoculation and give rise to infections such as lung infection, septicemia, chronic bronchitis and brain abscess [3–7] Patients with immunodeficiency, AIDS, cancer and diabetes, connective tissue disorders, and those who receive transplants or immunosuppressive drugs are among the main hosts of *Nocardia* infections [7]. However, isolated cases have been reported in immunocompetent hosts [8–11].

Microbiological isolation and identification of *Nocardia* from clinical samples by the conventional methods in general hospital laboratories are difficult. Molecular methods have proven to be highly discriminatory. These methods are currently in use in research and the reference laboratories as evidenced in the literature on *Nocardia* [12, 13]. The potential for incorporation of molecular techniques in clinical microbiology for quicker and more accurate species identification is fundamental for determination of treatment plans and prediction of antimicrobial sensitivity [12].

### Patient history

**Fig. 1.**
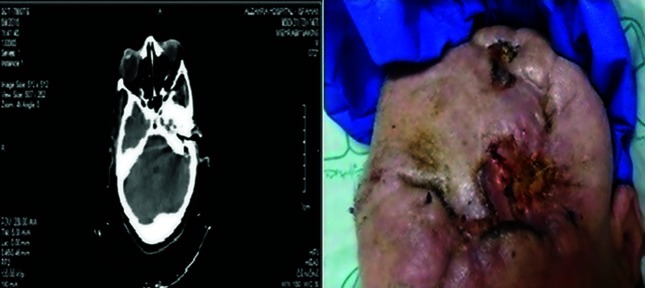
CT scan (left window) and the surgical site on the patient’s head (right window).

The patient was a 73-year-old woman admitted to hospital in May 2015 due to severe headache and shortness of breath. The patient suffered from diabetic foot ulcer, fever and vomiting at the time of hospitalization. She also had deep venous thrombosis (DVT) in the lower extremities. The lower respiratory tract infection was not confirmed by chest X-ray on her first hospital admission. The patient was initially suspected to have central nervous system (CNS) infection, and in the course of her treatment additional diagnosis of brain abscess was made and ultimately diagnosis of metastatic carcinoma was also histologically confirmed. The laboratory information of the patient is listed in Table 1. The medical examination and computed tomography (CT) detected a metastatic carcinoma involving skull bone in the tumor. In microscopic examination of the cerebral mass, neoplastic proliferation of epithelial cells was observed as cellular plates. Most cells were hyperchromatic or vesicular and nucleated. The patient's condition resulted in two surgical operations in the following two years (Fig. 1). However, no chemotherapy was used.

**Table 1.** Laboratory test results of the patient

Laboratory tests											
	FBS (mg dl^−1^)	WBC (Mc l^−1^)	BUN (mg dl^−1^)	CRP (mg l^−1^)	ESR (mm h^−1^)	K (mEq l^−1^)	Na (mEq l^−1^)	LDH (U l^−1^)	PTT (S)	Hb (g dl^−1^)	Cr (mg dl^−1^)
First surgery	153	15 000	14	83	72	4.2	135	741	49	10.4	0.8
Second surgery	130	6500	7	58	54	4.3	137	–	34	11.6	0.7

*FBS, fasting blood sugar; WBC, white blood cells; BUN, blood urea nitrogen; CRP, C-reactive protein; ESR, erythrocyte sedimentation rate; K, potassium; Na, sodium; LDH, lactic acid dehydrogenase; PTT, partial thromboplastic time; Hb, hemoglobin; Cr, creatinine.

## Methods

After the first surgery, in addition to general laboratory tests, brain biopsy and pathological examination of the presenting lesion was undertaken by the hospital general laboratory. The biopsy was investigated by direct microscopy test.

In the second surgery isolates from the diabetic foot ulcer and the brain biopsy taken from the patient were investigated by our research laboratory. The foot ulcer isolates were identified by culture and phenotypic features and the isolate from the brain biopsy was identified by conventional microbiological tests that included direct smear analysis by Gram and partial acid-fast staining, culture on blood agar and Sauton’s agar and incubation at 37 and 45 °C. The biochemical assays used to identify the isolate included casein, tyrosine, xanthine, gelatin, aesculin and adenine degradations and urease and citrate production. The drug sensitivity of the isolate was evaluated by the broth microdilution method, based on criteria indicated in CLSI M24-A2 [14]. The molecular identification of the isolate included the PCR-based amplification of a target genetic marker i.e. a 596 bp fragment of the 16S rRNA gene as recommended by Laurent *et al.* [15] confirming the identity of the organism at the genus level followed by 16S rRNA sequencing for definitive identification at the species level [16].

## Results

**Fig. 2.**
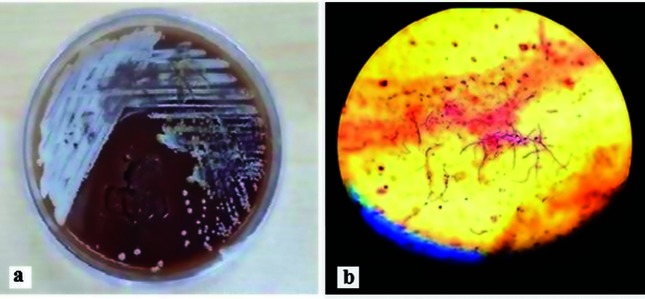
a; Small chalky white non-hemolytic colonies of the isolate on blood agar medium. b; Microscopic morphology of the isolate; filamentous branching Gram-positive rods (×1000).

After the first brain surgery the brain biopsy was examined by the hospital laboratory and, based on the results from direct microscopy tests, *Candida* sp. was reported. After the second surgery the clinical samples investigated by our research laboratory. *Staphylococcus aureus* and *Escherichia coli* were isolated from the foot ulcer and confirmed by conventional microbiological tests. The results of the brain biopsy investigation by microbiological tests revealed growth of chalky bacterial colonies with a soil aroma on culture media (Fig. 2). The Gram and partial acid-fast staining of the colonies indicated Gram-positive rods with branched filaments resembling species of the genus *Nocardia* (Fig. 2). The organism was able to grow at 37 and 45 °C and was positive for catalase production, aesculin hydrolysis and sucrose fermentation and was negative for casein, tyrosine, citrate, xanthine, gelatin and urease assays. The antibiotic sensitivity test showed that the isolate was sensitive to trimethoprim–sulfamethoxazole, doxycycline, amikacin, ciprofloxacin, penicillin G and metronidazole, had intermediate resistance to ceftriaxone and erythromycin and was resistant to imipenem and vancomycin.

The PCR amplification of a genetic marker based on a 596 bp region of the 16S rRNA confirmed the identity of the isolate as a member of the genus *Nocardia*. The 16S rRNA gene sequence of the isolate (937 base pairs) showed 100 % similarities with the corresponding sequence of *Nocardia cyriacigeorgica* strain ATCC 14759. The GenBank accession number of the 16S rRNA sequence of the Iranian isolate is MF437315.

## Discussion

Nocardiosis is an acute, sub-acute, or chronic infectious disease that occurs in cutaneous, pulmonary and disseminated forms. Primary cutaneous nocardiosis manifests as cutaneous infection (cellulitis or abscess), lymphocutaneous infection (Sporotrichoid nocardiosis), or subcutaneous infection (actinomycetoma). Pleuropulmonary nocardiosis manifests as an acute, sub-acute, or chronic pneumonitis, usually in immunocompromised hosts, although clinical cases have also been reported in immunocompetent hosts. Disseminated nocardiosis may involve any organ; however, lesions in the brain or meninges are most common. The first report of nocardiosis due to a novel species of the genus *Nocardia*, i.e., *Nocardia cyriacigeorgica,* was reported by Yasin *et al.* in 2001. The organism was isolated from a chronic bronchitis patient [2]. Since then, *Nocardia cyriacigeorgica* has been reported as a newly-emerging pathogen isolated from clinical cases in many regions of the world [5, 17].

In a study in 2005, *Nocardia cyriacigeorgica* was isolated from a brain abscess patient with AIDS [7]. In another study in 2006, two cases of septicemia by *Nocardia cyriacigeorgica* were reported [4]. In a study by Schlaerg *et al.* in 2008, the first case of lung infection by *Nocardia cyriacigeorgica* was reported [5]. In 2011, Shojaei *et al.* reported the clinical isolation of *Nocardia cyriacigeorgica* from Iranian patients with different clinical symptoms [18]. In another study from Turkey, in 2014, *Nocardia cyriacigeorgica* was isolated from brain abscess of two patients with multiple myeloma [6].

The present study shows that *Nocardia* infection, due to lack of specific clinical manifestations, can be easily misdiagnosed as fungal or tuberculosis infection, invasive fungal disease and malignancy, as was the case in the studied Iranian patient. Isolation and identification of *Nocardia* strains is considered the only reliable diagnostic method [19].

The rare infections, such as brain abscess, due to fastidious organisms, such as *Nocardia*, in patients with underlying diseases are easily neglected in the developing countries. This is the results of inadequate microbiological experience of laboratory technicians or lack of standard microbiological protocols to consider and screen the rare fastidious microorganisms.

The presumptive identification of nocardia can be achieved on the basis of macroscopic and microscopic morphology, resistance to lysozyme and restriction profiles using the PRA-*hsp*65 method. Isolates with characteristics of filamentous bacilli, forming aerial hyphae, with colonies that may be pigmented, rough and without the *Bst*EII digestion pattern in PRA-*hsp*65 method are suggestive of *Nocardia.* The PRA-*hsp*65 method [13, 14] is routinely used to amplify a 440 bp gene fragment that encodes a protein of 65 kDa for mycobacteria and actinomycetes [15]. As *Nocardia* lack the *Bst*EII restriction site, this feature can be used in the presumptive identification of the genus. Additionally, *Nocardia* PRA-*hsp*65 patterns with HaeIII are different to the algorithm described by Roth *et al.* [16]. When necessary, sequencing of the 16S rRNA gene provides an alternative identification method for the rapid identification of *Nocardia* species of clinical significance which is even more sensitive than restriction endonuclease analysis (REA) of the HSP gene [17].

Our study underscored growing concerns over misdiagnosis of the emerging pathogens and in particular those that are hard to detect in routine hospital laboratories in a developing country. This fact, in turn, indicates requirements for quality and competence in medical laboratories in order to improve and incorporate microbiological diagnostic procedures for isolation and identification of emerging pathogens, such as mycobacteria and nocardia.
